# Supplementary material for: The Mn-motif protein MAP6d1 assembles ciliary doublet microtubules
Source: Nat Commun. 2025 Jul 5;16:6210. doi: 10.1038/s41467-025-61679-0 (PMC12228683; doi:10.1038/s41467-025-61679-0)
Supplement: Supplementary file 1 — Supplementary Information [file 41467_2025_61679_MOESM1_ESM.pdf]

Supplementary Information

The Mn-motif protein MAP6d1 assembles ciliary doublet microtubules

Dharshini Gopal[1#], Juliette Wu[1#], Julie Delaroche[1], Christophe Bosc[1], Manon De Andrade[1], Eric Denarier[1], Gregory Effantin[2], Annie Andrieux[1], Sylvie Gory-Fauré[1*], Laurence Serre[1‡*], Isabelle Arnal[1‡*]

[1]Université Grenoble Alpes, INSERM, U1216, CNRS, CEA, Grenoble Institut Neurosciences (GIN), Grenoble, France.

[2]Institut de Biologie Structurale (IBS), Université Grenoble Alpes, CNRS, CEA, 71 Avenue des Martyrs, 38042 Grenoble, France.

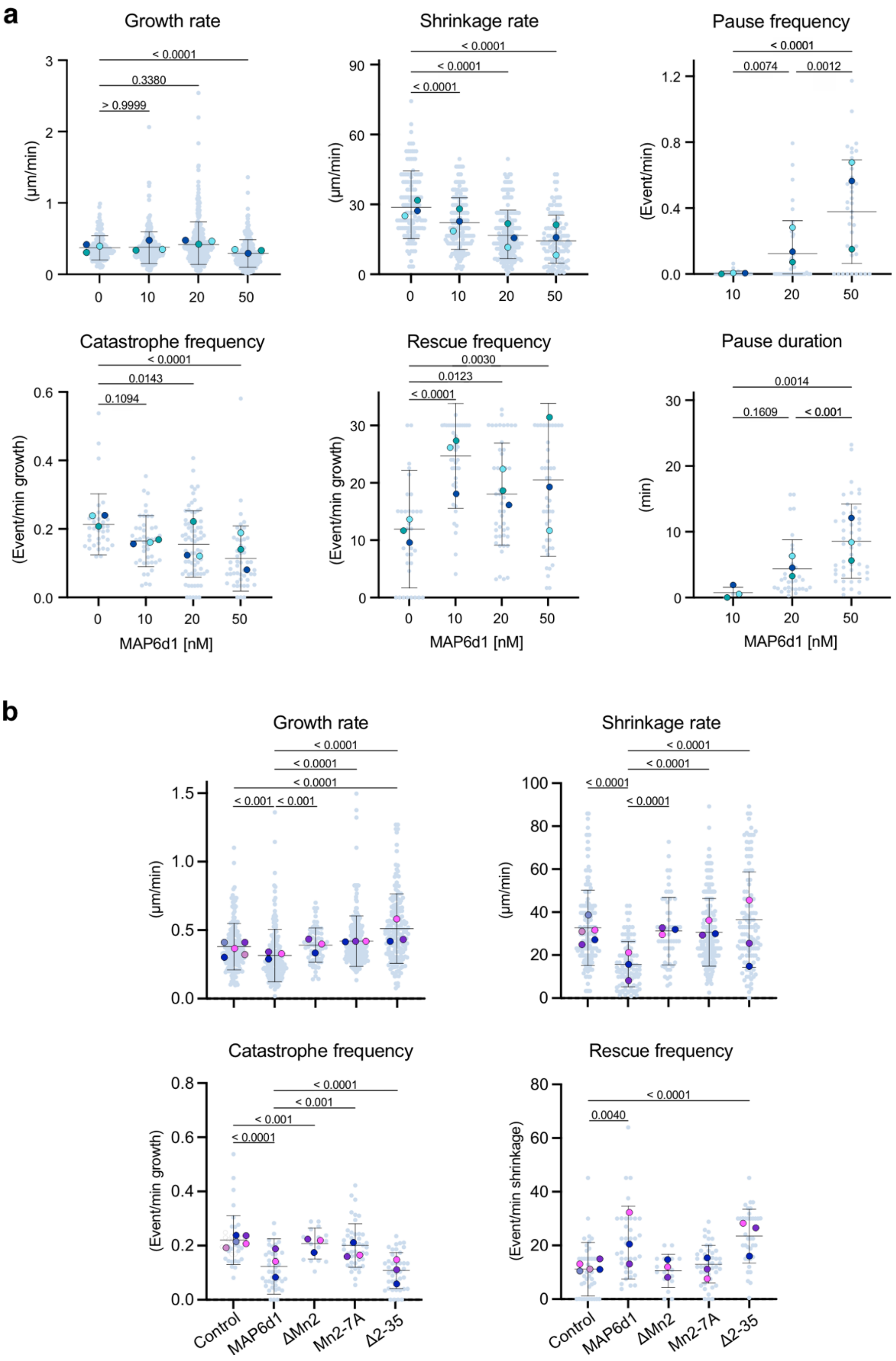

Supplementary Figure 1. Microtubule minus end dynamic modulation by MAP6d1 and its mutants.
a Graphs showing the growth rate (n = 158, 266, 361, and 217 events for 0, 10, 20 and 50 nM MAP6d1, respectively), shrinkage rate (n = 133, 182, 202 and 116 events), catastrophe and rescue frequencies (n =

35, 43, 72 and 51 MTs), the pause frequencies (n = 31, 41 and 39 MTs, for 10, 20 and 50 nM MAP6d1, respectively) and pause durations (n = 4, 34 and 49 events) of MTs assembled with increasing concentrations of MAP6d1, extracted from kymographs depicted in **Fig. 1a**. **b** Dynamical parameters of microtubules assembled with tubulin alone and in the presence of MAP6d1, MAP6d1-ΔMn2, MAP6d1-Mn2-7A and MAP6d1-Δ2-35: growth rate (n = 187, 175, 68, 239 and 184 growth events, respectively), shrinkage rate (n = 162, 98, 56, 205 and 117 shrinkage events, respectively), catastrophe and rescue frequencies (n = 39, 41, 18, 46 and 41 frequencies), extracted from kymographs depicted in **Fig. 3a**. Bars represent mean ± SD from at least three independent experiments. Circles with different colours represent the mean of each individual experiment. p values are indicated (Kruskal-Wallis ANOVA followed by post hoc Dunn's multiple comparison test. For clarity, only significant statistics are indicated for (**b**). Source data are provided as a Source Data file.

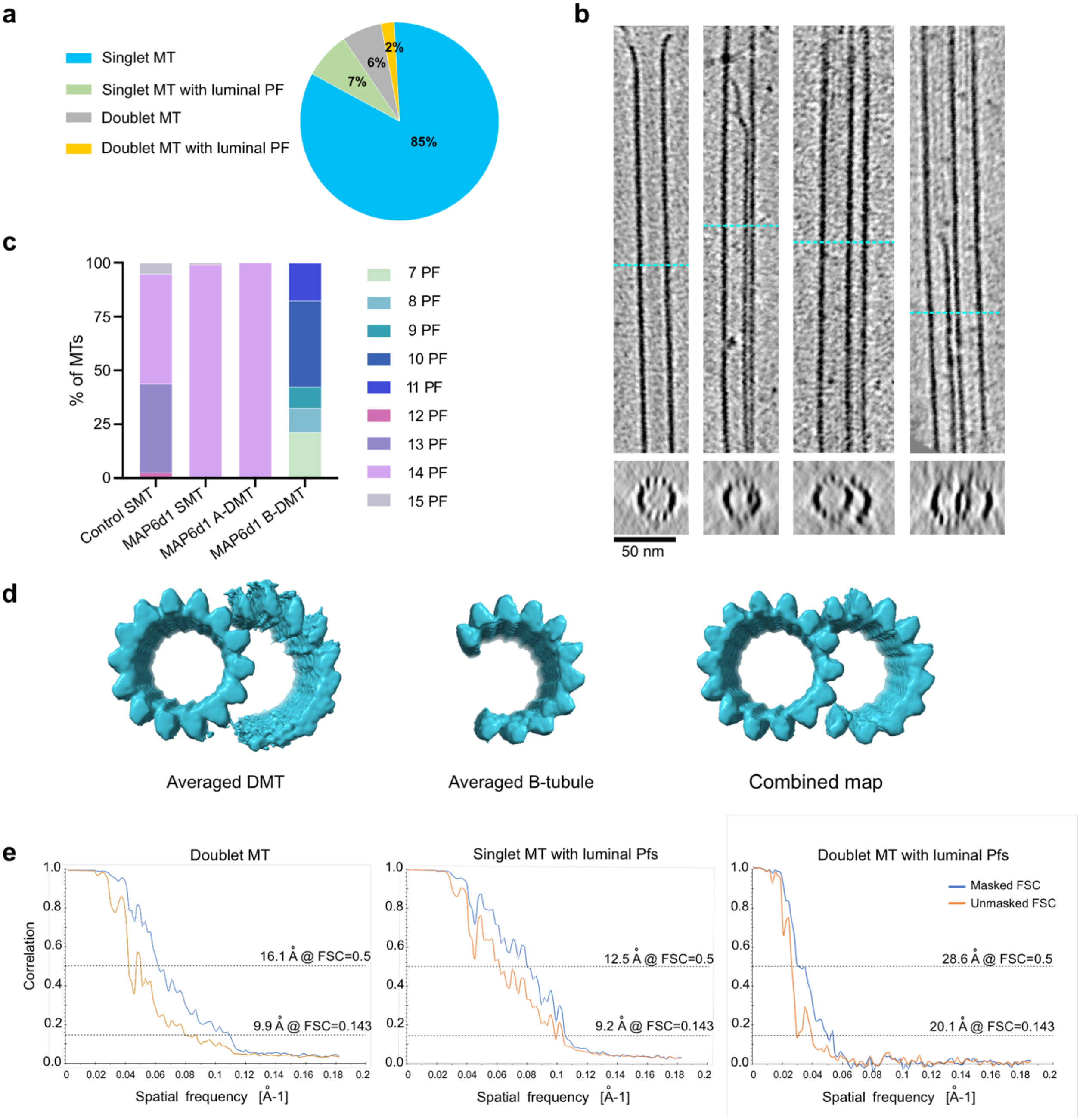

Supplementary Figure 2. Analysis of MAP6d1-induced microtubule architectures. a Distribution of microtubule architectures in the tomography data set. **b** Examples of different microtubule architectures induced by MAP6d1. Blue dashed lines indicate the location at which the z-sections are displayed. **c** Distribution of microtubules (MT) with different number of protofilaments (PF) measured along 265 μm of microtubule length for control, and for microtubules polymerised by MAP6d1 are 820 and 115 μm of singlet (SMT) and doublet (DMT) microtubule lengths, respectively. **d** Subtomogram averaged models of the initial doublet microtubule, B-tubule and combined map. **e** Fourier Shell Correlation (FSC) plots of

reconstructions shown in this study obtained from Eman2 refinement output. Source data are provided as a Source Data file.

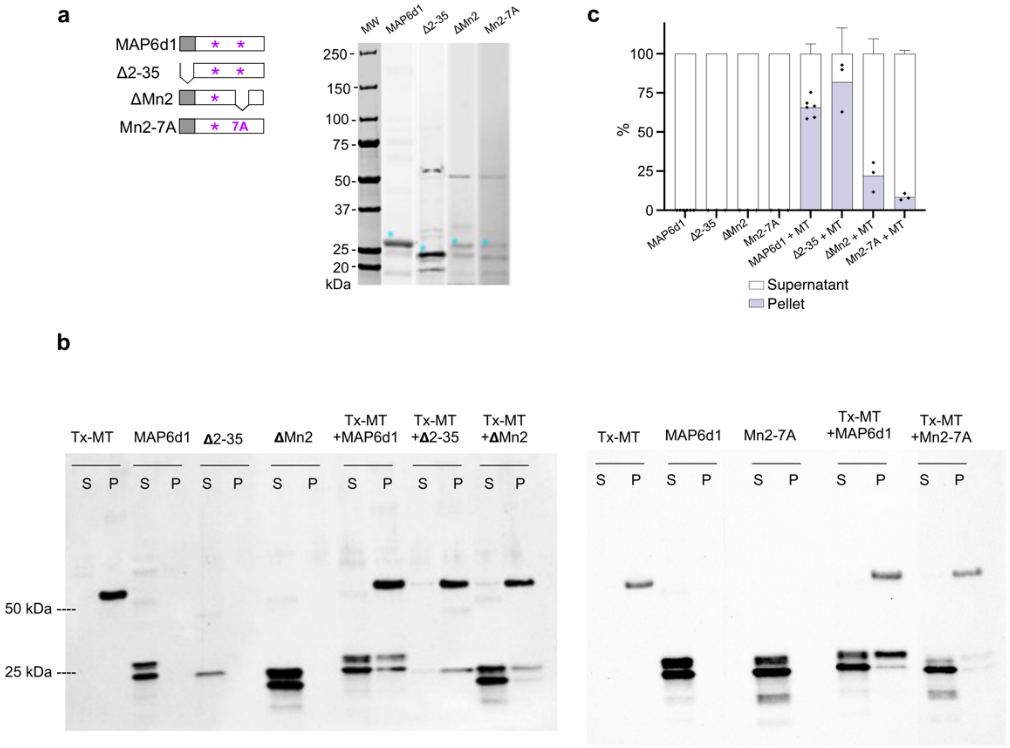

Supplementary Figure 3. Purified recombinant proteins used in this study and their microtubule-binding properties. a Purified MAP6d1 and the mutants (cyan asterisk) on Coomassie-blue-stained gels (10 % SDS PAGE). To the left are diagrams of full-length MAP6d1 and the mutants with the N-terminal in grey and the Mn-motifs with a pink asterisk. MW, molecular weight. **b** Representative immunoblots of co-sedimentation assays of Taxol-stabilised microtubules (Tx-MT) with MAP6d1, and the mutants detected with antibodies against tubulin and histidine-tag (to detect his-tag purified MAP6d1 and mutants). S-Supernatant; P-Pellet. **c** Analysis of blots presented in (**b)**. Black dots represent the percentage of proteins in the pellet for each blot. Source data are provided as a Source Data file.

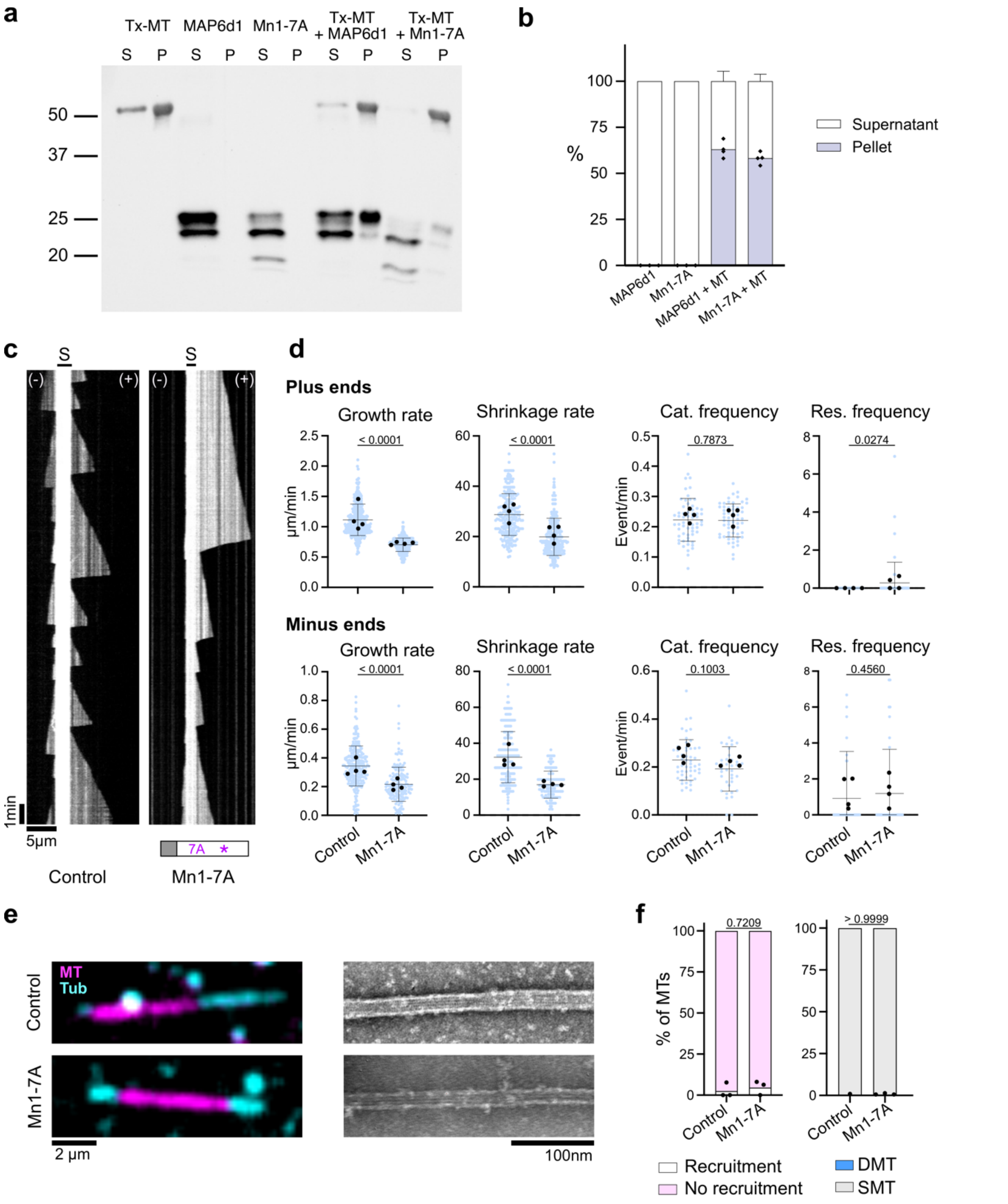

Supplementary Figure 4. Effects of the mutant MAP6d1-Mn1-7A on microtubule dynamics, tubulin recruitment and doublet microtubule formation. a Representative immunoblots of co-sedimentation assays of Taxol-stabilised microtubules (Tx-MT) with MAP6d1-Mn1-7A, detected with antibodies against tubulin and histidine-tag (to detect his-tag purified MAP6d1 mutant). S-Supernatant; P-Pellet. **b** Analysis of blots presented in (**a**). Black dots represent the percentage of proteins in the pellet for each blot. **c** Representative kymographs of microtubules grown from seeds (S) and 12 μM tubulin alone or with

300 nM MAP6d1-Mn1-7A. **d** Dynamical parameters of microtubules assembled with tubulin alone or in the presence of MAP6d1-Mn1-7A. Plus end: growth rate (n = 323 and 319 events, respectively), shrinkage rate (n = 233 and 274 events, respectively), catastrophe and rescue frequencies (n = 60 and 62 microtubules, respectively). Minus end: growth rate (n = 243 and 152 events, respectively), shrinkage rate (n = 201 and 130 events, respectively), catastrophe and rescue frequencies (n = 52 and 38 microtubules, respectively). Bars represent mean ± SD from at least three independent experiments. Circles represent the mean of each individual experiment. p values are indicated (Kruskal-Wallis ANOVA followed by post hoc Dunn's multiple comparison test). **e** *Left*: snapshots of TIRF movies of 0.325 μM tubulin (cyan) mixed with GMPCPP seeds (magenta) in the absence (Control) or presence of 300 nM MAP6d1-Mn1-7A. *Right*: Negative staining electron microscopy images of microtubules assembled in the same conditions as for TIRF microscopy. MT, microtubule; Tub, tubulin. **f** *Left*: Percentage of microtubules with or without recruited tubulin in the control and with MAP6d1-Mn1-7A (87 and 117 microtubules for the control and with MAP6d1-Mn1-7A, respectively). Black dots represent the percentage of microtubules with recruitment for each experiment. *Right:* percentage of singlet (SMT) and doublet (DMT) microtubules in the control and with MAP6d1-Mn1-7A (total measured lengths of 195 and 409 µm, respectively). Black dots represent the percentage of doublet microtubules for each experiment. p values are indicated (two-sided Fischer's exact contingency test). Source data are provided as a Source Data file.

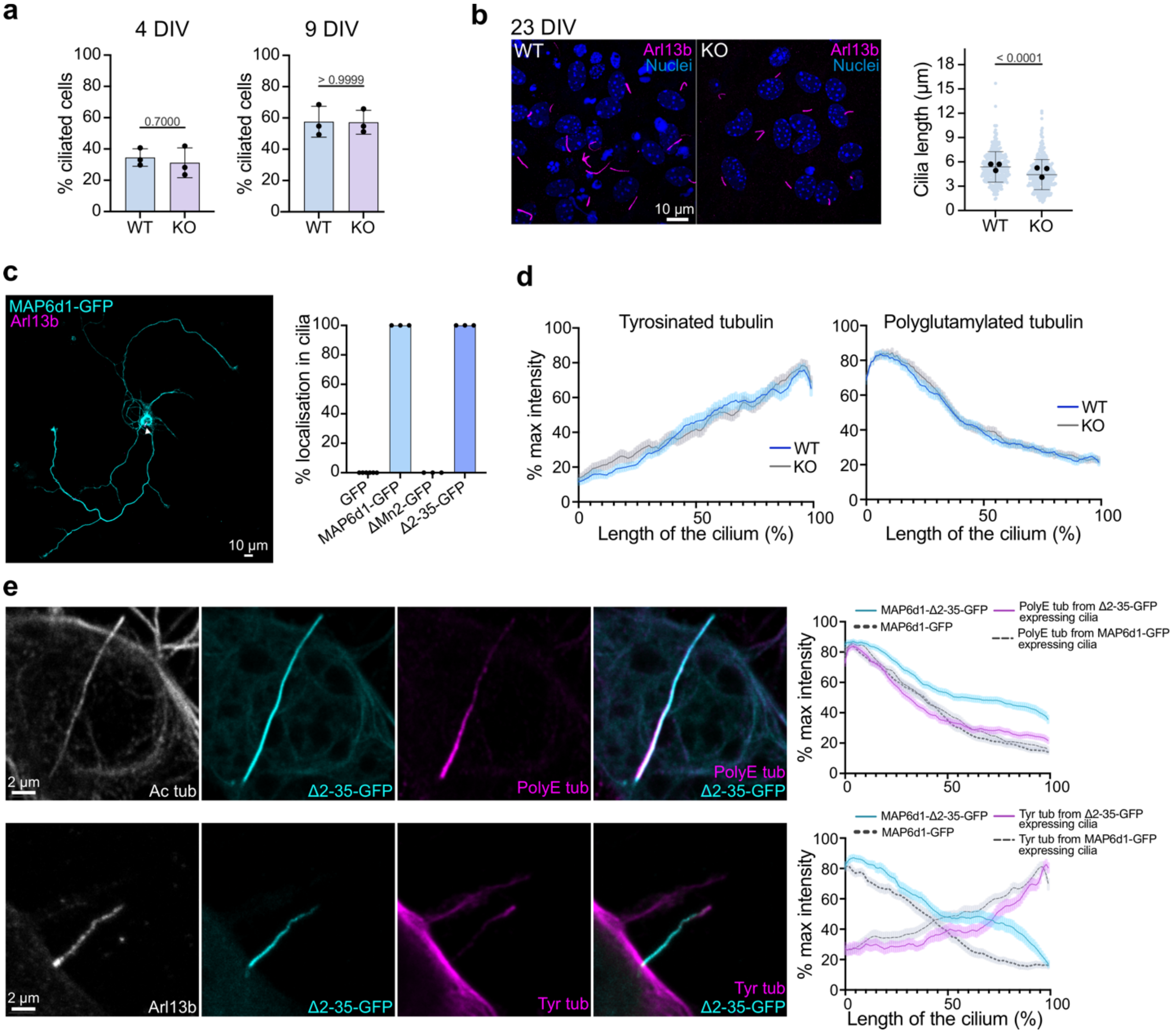

Supplementary Figure 5. Comparison of primary cilia from wildtype and MAP6d1 deficient neurons. a Quantification of ciliated cells in hippocampal neurons at 4 (*left*) and 9 days *in vitro* (DIV) (*right*). n $\geq$ 350 cells for each experiment. Bars represent mean ± SD from three independent experiments. Circles represent the ratio of ciliated cells of each experiment. p values are indicated (Mann-Whitney's test). **b** Representative images of wild-type (WT) and MAP6d1-knockout (KO) hippocampal neurons at 23 DIV stained for Arl13b and Hoechst. Quantification of cilium length from WT (n = 344 cilia) and KO neurons (n = 385 cilia) (3 experiments). Bars represent mean ± SD and circles, the mean of each experiment. p value is indicated (Mann-Whitney's test). **c** Hippocampal neuron at 7 DIV transfected with MAP6d1-GFP and stained for Arl13b. White arrow indicates the primary cilium. The graph represents the proportion of localisation to the cilia in neurons transfected with plasmids encoding GFP (n = 128 neurons), MAP6d1-GFP (n = 68 neurons), MAP6d1-ΔMn2-GFP (n = 59 neurons), MAP6d1-Δ2-35-GFP

(n = 86 neurons) from at least three independent experiments. **d** *Left:* Normalised maximum fluorescence of tyrosinated tubulin along the ciliary length determined by Arl13b in 9 DIV WT (n = 42 cilia) and MAP6d1-KO (n = 47 cilia) hippocampal neurons. *Right:* Normalised maximum fluorescence of polyglutamylated tubulin along the ciliary length determined by acetylated tubulin in 9 DIV WT (n = 45 cilia) and MAP6d1-KO (n = 47 cilia) hippocampal neurons. Values represent mean ± SEM from 3 independent experiments. **e** *Top*: Representative images of primary cilia from 7 DIV hippocampal neurons expressing MAP6d1-Δ2-35-GFP stained for acetylated tubulin (Ac tub) and polyglutamylated tubulin (PolyE tub). Solid lines show normalised maximum fluorescence of MAP6d1-Δ2-35-GFP and polyglutamylated tubulin (PolyE tub) along the ciliary length determined by acetylated tubulin. Values represent mean ± SEM of n = 94 cilia from 3 independent experiments. Dotted lines represent normalised maximum fluorescence of MAP6d1-GFP and polyglutamylated tubulin (PolyE tub) along the ciliary length in 7 DIV hippocampal neurons expressing ectopic MAP6d1-GFP (data from **Fig. 4f**). *Bottom*: Representative images of primary cilia from 7 DIV hippocampal neurons expressing MAP6d1-Δ2-35-GFP stained for Arl13b and tyrosinated tubulin (Tyr tub). Plain lines show normalised maximum fluorescence of MAP6d1-Δ2-35-GFP and tyrosinated tubulin (Tyr tub) along the ciliary length determined by Arl13b. Values represent mean ± SEM of n = 35 cilia from 3 independent experiments. Dotted lines show normalised maximum fluorescence of MAP6d1-GFP and tyrosinated tubulin (Tyr tub) along the ciliary length in 7 DIV hippocampal neurons expressing ectopic MAP6d1-GFP (**Fig. 4g**). Source data are provided as a Source Data file.

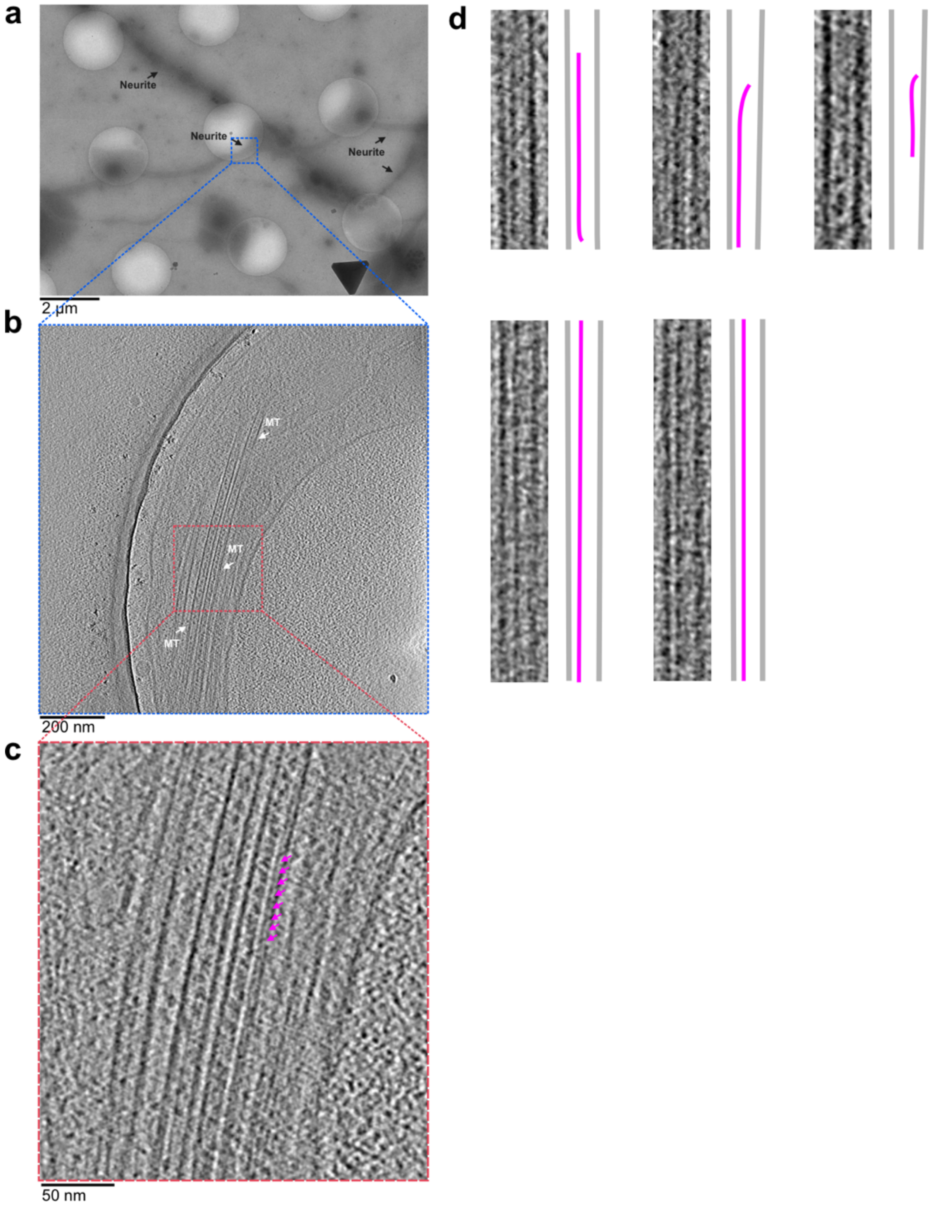

Supplementary Figure 6. Cryo-electron tomography of microtubules in neurons. a Low-magnification images of mature neurons on EM grids acquired using Titan Krios cryo-electron microscope. White arrows indicate neurites. **b** High-magnification tomogram of neuritic extensions highlighting microtubules (MTs) with white arrows. **c** Zoomed-in image of the neuronal microtubule with luminal protofilaments indicated by cyan arrows. **d** Gallery of images showing filamentous densities resembling luminal protofilaments in singlet neuronal microtubules, and their corresponding schemes.

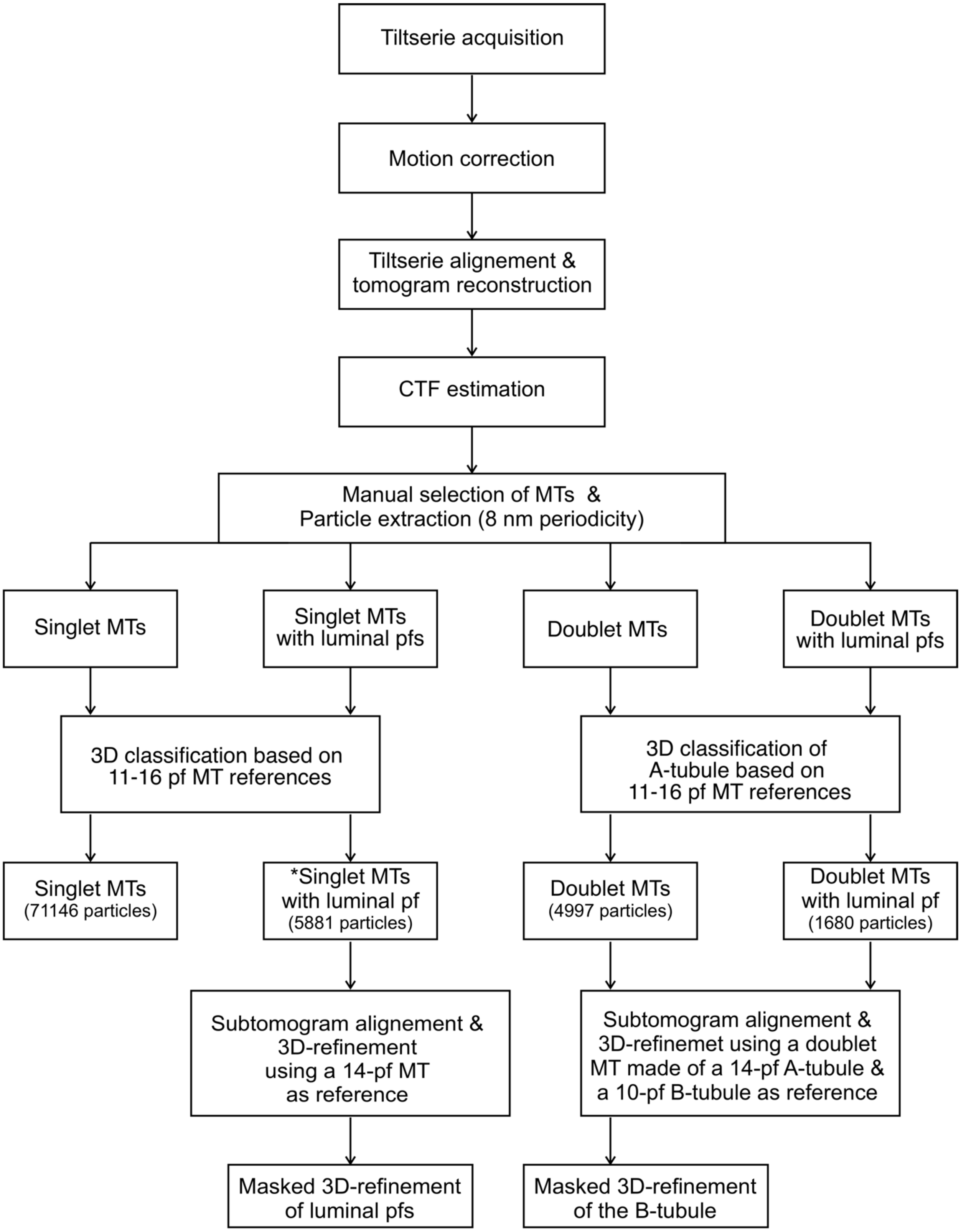

* All singlet MTs with luminal pfs were classified as 14-pf MTs with two luminal pfs.

Supplementary Figure 7. Cryo-EM processing workflow. MT, microtubule; pf, protofilament.

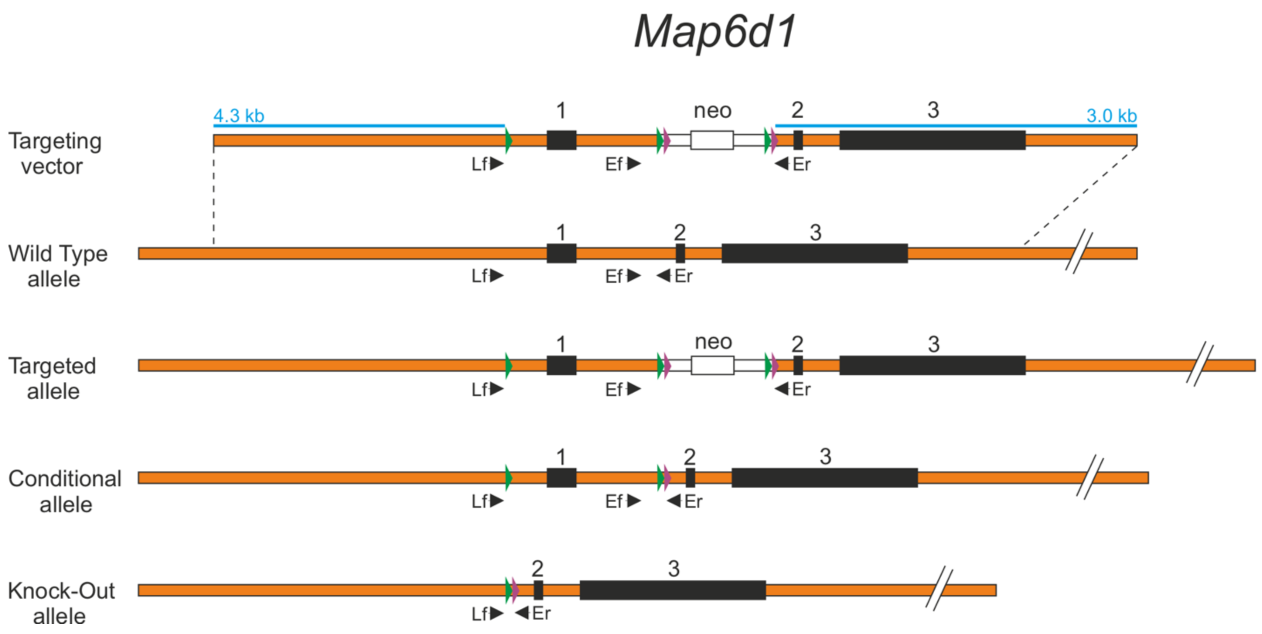

Supplementary Figure 8. MAP6d1-KO mice generation and genotyping strategy. Diagram of the targeting vector used and all the possible alleles for *MAP6d1*, and of the wild-type and knock-out alleles for *MAP6d1* (bottom). Orange bar: genomic DNA. Black box: exons with their corresponding number. Green and purple arrowheads: LoxP and Flp sequences, respectively. White bar: neo cassette, with the neomycin resistance gene (white box). Blue lines: zone of sequence homology for homologous recombination, with the corresponding size in kbp. Black arrowheads: primers used for the PCR genotyping.

Plasmid	Sequence	PCR Matrix	Cloning Vector
MAP6d1	For 5'-AAGGAGATATACATATGGCGTGGCCCTGCATCAG-3'	Map6d1-EGFP	pET42a (Nde I / Xho I) Novagen
	Rev 5'-GGTGGTGGTGCTCGAGTGCGGCCGCTCAATGGTGATGGTGATGATGGTGATGGGTGGCGACCGGTGGATCC-3'		
MAP6d1 -Δ2-35	For 5'-AAGGAGATATACATATGGGGAGCGAGGAGTCGGGC-3'	Map6d1-EGFP	pET42a (Nde I / Xho I) Novagen
	Rev 5'-GGTGGTGGTGCTCGAGTGCGGCCGCTCAATGGTGATGGTGATGATGGTGATGGGTGGCGACCGGTGGATCC-3'		
MAP6d1 -ΔMn2	For 5'-AAGGAGATATACATATGGCGTGGCCCTGCATCAG-3'	MAP6d1 -ΔMn3	MAP6d1 + pET42a (Nde I / BamH I) This work
	Rev 5'-GGCGACCGGTGGATCCCCCACATTGAGAGTCTGAGGAGC-3'		
MAP6d1 -Mn2-7A	For 5'-AAGGAGATATACATATGGCGTGGCCCTGCATCAG-3'	Map6d1-EGFP	MAP6d1 + pET42a (Nde I / Hind III) This work
	Rev 5'-ACTCCAGTCCAAGCTTGCGCTGCCGCAGCCGCGGCCGCGGTCGCTACTACAGCTGCGTC-3'		

Supplementary Table 1. Sequence of the primers used to produce plasmids encoding MAP6d1 and its mutants. For each construct, the sequence of the Forward (For) and Reverse (Rev) primers are indicated, along with the matrix used to produce the PCR fragments and the restriction enzymes used to linearize the cloning vector. All PCR matrices are from ref (30).

Name	SMT with luminal pfs	DMT with luminal pfs	DMT	luminal pfs in SMT	B-tubule in DMT
EMDB code	EMD-52569	EMD-52572	EMD-52575	EMD-53452	EMD-52574
Magnification	33,000 X (K3 Gatan camera)				
Voltage	300 kV (Titan Krios)				
Total electron dose	117 electrons /Å^2				
Defocus range	-3 to -6 μm				
Pixel size	2.7 Å				
Symmetry imposed	C1				
Subtomogram number	5881	1680	4997	5881	4997
Map resolution masked/unmasked at FSC 0.5	12.5 / 16.7 Å	28.6 / 37.4 Å	16.1 / 20 Å	21 / 24.7 Å	21.7 / 23.8 Å

Supplementary Table 2. **Parameters for cryo-electron tomography data collection and processing.** FSC, Fourier Shell Correlation; DMT, doublet microtubule; SMT, singlet microtubule; pfs, protofilaments.

	Type of experiments	[Tubulin]	[MAP6d1] [mutants]	Figure	Results
**MAP6d1**	MT dynamics (TIRFm)	12 µM	10-50 nM	Fig 1a-c<br>Fig S1a	MT stabilization: reduced growth & shrinkage; increased rescues & pauses
	MT architectures (EM)	25 µM	250 nM	Fig 2a-d<br>Fig S2	DMT
	Tubulin recruitment on GMPCPP stabilized MTs (TIRFm & EM)	0.325 µM soluble tubulin	100 nM	Fig 2e-f	Tubulin recruitment and DMT
	Co-sedimentation with Taxol-stabilized MTs	1 µM	300 nM	Fig S3b-c	65% of MAP6d1 co-pelleted with MT
**Mn-motif mutants**	MT dynamics (TIRFm)	12 mM	300 nM	Fig 3a-b<br>Fig S1b<br>Fig S4c-d	No MT stabilization
	Tubulin recruitment on GMPCPP stabilized MTs (TIRFm & EM)	0.325 µM soluble tubulin	300 nM	Fig 3c-d<br>Fig S4e-f	No tubulin recruitment, no DMT
	Co-sedimentation with Taxol-stabilized MTs	1 µM	300 nM	Fig S3b-c<br>Fig S4a-b	<25% (Mn2-mutants) and 58% (Mn1 mutant) co-pelleted with MT
**Δ2-35 mutant**	MT dynamics (TIRFm)	12 µM	50 nM	Fig 3a-b<br>Fig S1b	MT stabilization: Increased growth rate and rescue frequency
	Tubulin recruitment on GMPCPP stabilized MTs (TIRFm & EM)	0.325 µM soluble tubulin	100 nM	Fig 3c-d	No tubulin recruitment and no DMT
	Co-sedimentation with Taxol-stabilized MTs	1 µM	300 nM	Fig S3	80% co-pelleted with MT

Supplementary Table 3. Overview of the *in vitro* techniques and protein concentrations used in this study and the main conclusions. DMT, doublet microtubule; MT, microtubule; EM, electron microscopy; TIRFm, TIRF microscopy.
