# Supplementary material for: The Mn-motif protein MAP6d1 assembles ciliary doublet microtubules
Source: Nat Commun. 2025 Jul 5;16:6210. doi: 10.1038/s41467-025-61679-0 (PMC12228683; doi:10.1038/s41467-025-61679-0)
Supplement: Supplementary file 2 — Description of Additional Supplementary Files [file 41467_2025_61679_MOESM2_ESM.pdf]

Description of Additional Supplementary Files

File Name: Supplementary Movie 1

Description: Assessment of microtubule dynamics. TIRF microscopy captures microtubules growing from seeds in the presence of fluorescent GTP-tubulin (12 µM) without (control) and with 50 nM MAP6d1 or Δ2-35-MAP6d1 (Delta2-35). The green asterisk highlights the paused microtubule in the presence of MAP6d1. Scale bar, 2 µm.

File Name: Supplementary Movie 2

Description: Tubulin recruitment assay. TIRF microscopy shows microtubule seeds (magenta) in the presence of 325 nM tubulin (cyan) and 50 µM GMPCPP without (control) and with 100 nM MAP6d1 or Δ2-35-MAP6d1 (Delta2-35). The white asterisks indicate examples of recruited tubulin on the microtubule seed in the presence of MAP6d1. Scale bar, 2 µm.
